# Emergence and evolution of antimicrobial resistance genes and mutations in *Neisseria gonorrhoeae*

**DOI:** 10.1186/s13073-021-00860-8

**Published:** 2021-03-30

**Authors:** Koji Yahara, Kevin C. Ma, Tatum D. Mortimer, Ken Shimuta, Shu-ichi Nakayama, Aki Hirabayashi, Masato Suzuki, Michio Jinnai, Hitomi Ohya, Toshiro Kuroki, Yuko Watanabe, Mitsuru Yasuda, Takashi Deguchi, Vegard Eldholm, Odile B. Harrison, Martin C. J. Maiden, Yonatan H. Grad, Makoto Ohnishi

**Affiliations:** 1 Antimicrobial Resistance Research Center, National Institute of Infectious Diseases, Tokyo, Japan; 2 Department of Immunology and Infectious Diseases, Harvard T. H. Chan School of Public Health, Boston, MA USA; 3 Department of Bacteriology I, National Institute of Infectious Diseases, Tokyo, Japan; 4 Department of Microbiology, Kanagawa Prefectural Institute of Public Health, Chigasaki, Kanagawa Japan; 5 Present address: Faculty of Veterinary Medicine, Okayama University of Science, 1-3 Ikoinooka, Imabari, Ehime 794-8555 Japan; 6 Center for Nutrition Support and Infection Control, Gifu University Hospital, Gifu, Japan; 7 Department of Urology, Kizawa Memorial Hospital, Gifu, Japan; 8 Division of Infection Control and Environmental Health, Norwegian Institute of Public Health, Oslo, Norway; 9 Department of Zoology, University of Oxford, Oxford, UK

**Keywords:** Recombination, Horizontal gene transfer, Genomic epidemiology, Antimicrobial resistance, Phylogeny, Surveillance, Evolution, *Neisseria gonorrhoeae*

## Abstract

**Background:**

Antimicrobial resistance in *Neisseria gonorrhoeae* is a global health concern. Strains from two internationally circulating sequence types, ST-7363 and ST-1901, have acquired resistance to third-generation cephalosporins, mainly due to mosaic *penA* alleles. These two STs were first detected in Japan; however, the timeline, mechanism, and process of emergence and spread of these mosaic *penA* alleles to other countries remain unknown.

**Methods:**

We studied the evolution of *penA* alleles by obtaining the complete genomes from three Japanese ST-1901 clinical isolates harboring mosaic *penA* allele 34 (*penA-*34) dating from 2005 and generating a phylogenetic representation of 1075 strains sampled from 35 countries. We also sequenced the genomes of 103 Japanese ST-7363 *N. gonorrhoeae* isolates from 1996 to 2005 and reconstructed a phylogeny including 88 previously sequenced genomes.

**Results:**

Based on an estimate of the time-of-emergence of ST-1901 (harboring mosaic *penA-34*) and ST-7363 (harboring mosaic *penA*-10), and > 300 additional genome sequences of Japanese strains representing multiple STs isolated in 1996–2015, we suggest that *penA*-34 in ST-1901 was generated from *penA*-10 via recombination with another *Neisseria* species, followed by recombination with a gonococcal strain harboring wildtype *penA*-1. Following the acquisition of *penA*-10 in ST-7363, a dominant sub-lineage rapidly acquired fluoroquinolone resistance mutations at GyrA 95 and ParC 87-88, by independent mutations rather than horizontal gene transfer. Data in the literature suggest that the emergence of these resistance determinants may reflect selection from the standard treatment regimens in Japan at that time.

**Conclusions:**

Our findings highlight how antibiotic use and recombination across and within *Neisseria* species intersect in driving the emergence and spread of drug-resistant gonorrhea.

**Supplementary Information:**

The online version contains supplementary material available at 10.1186/s13073-021-00860-8.

## Background

Antimicrobial resistance (AMR) is one of the greatest threats to human health and urgently requires effective global surveillance [1]. Gonorrhea is one of the most common sexually transmitted bacterial infections worldwide causing substantial morbidity and economic loss [2–4]. The resistance of *Neisseria gonorrhoeae* to third-generation cephalosporins (3GCs) and fluoroquinolones has been defined as “high” priority by the World Health Organization (WHO). Among 3GCs, cefixime is no longer recommended for single-dose treatments, whereas ceftriaxone is the only drug allowed for empirical first-line monotherapy in most countries [5]. Reduced susceptibility to cefixime and ceftriaxone is mainly caused by mutations in *penA*, which encodes the 3GC target penicillin-binding protein 2 (PBP2). Mosaic *penA* alleles are deemed “mosaic” as the 3′ segments of their DNA sequences have been imported from other *Neisseria* spp*.* via homologous recombination events [6]. Many mosaic alleles have been documented, reflecting both distinct recombination events and additional mutations [7], and many of these promote resistance to 3GCs.

Two internationally disseminated sequence types (STs) defined by *Neisseria* multilocus sequence typing (MLST), ST-7363 and ST-1901 (predominantly corresponding with NG-MAST types 1407 and 4220), have acquired mosaic *penA* alleles and can exhibit resistance to 3GCs [8]. ST-1901, harboring mosaic *penA*-34, accounted for the majority of isolates with reduced susceptibility to 3GCs in the USA and Europe from the 2000s to at least the early 2010s [9, 10]. In Japan, ST-7363 accounted for the highest proportion (66%) of cases among 149 isolates with reduced susceptibility to cefixime, isolated from 1998 to 2005 [8], and for 21% cases among 90 isolates obtained in 2015 [11]. These two STs harbor the mosaic *penA* alleles 34 and 10, which have identical nucleotide and amino acid sequences, except for those at the C-terminus (encoding 33 amino acids; approximately 6% of the entire sequence) [7, 11]; both STs likely originated in Japan [12]. Recently, we analyzed whole genome sequence data combined with antimicrobial susceptibility testing results from 204 isolates from genomic surveillance of a region where the first extensively drug resistant (XDR), ceftriaxone-resistant, *N. gonorrhoeae*, was isolated. This analysis was complemented with data from 67 genomes from other time frames (from 1996 to 2015) and locations within Japan [11]. Analysis of ST-1901-associated and ST-7363-associated core genome groups revealed distinct evolutionary pathways of mosaic *penA* acquisition: the ST-7363-associated core-genome group acquired *penA*-10 once, whereas the ST-1901-associated core-genome group had multiple independent acquisitions of *penA*-10 and *penA*-34. The previous study analyzed only Japanese *N. gonorrhoeae* isolates (mostly from 2015); thus, when and how the mosaic *penA* alleles—particularly *penA*-34, which is now dominant in USA and Europe—emerged and spread to other countries remains unknown. Although the generation of *penA*-10 was explained by a single horizontal gene transfer (HGT) or recombination event of the 3′ *penA* segment from a commensal *Neisseria* spp*.*, whether the generation of *penA*-34 is similarly explained by a single HGT event, or by multiple successive events, remains unknown. Furthermore, the distribution of fluoroquinolone-resistance determinants, i.e., mutations in *gyrA* and *parC* [10], was examined only in strains isolated in 2015; however, when and how those mutations emerged and spread is also unclear.

Here, we obtained complete, closed, genome sequences of three Japanese ST-1901 *N. gonorrhoeae* isolates harboring *penA*-34 from 2005 and reconstructed a dated phylogeny based on core-genome alignment of 1075 genome sequences from isolates sampled from 35 countries. We also sequenced the genomes of 103 Japanese ST-7363 *N. gonorrhoeae* isolates from 1996 to 2005 and reconstructed a dated phylogeny of these genomes and 88 previously sequenced Japanese ST-7363 genomes dating from 1996 to 2015. We further examined how these estimated dates corresponded to contemporary antibiotic use and treatment regimens. This generated a detailed narrative of the emergence and evolution of cephalosporin-resistance genes and fluoroquinolone-resistance mutations, based on the dated phylogenies of these two globally prevalent STs.

## Methods

Detailed descriptions of the methods used, including genome assembly, and construction of the clonal time-resolved phylogeny, followed by inference of the recombination events, and the associated results are provided in Additional file 1.

### Isolates and DNA sequencing

In total, 1075 genome sequences from *N. gonorrhoeae* isolates belonging to an ST-1901-associated core-genome group were included (Additional file 2: Table S1). This also comprised three mosaic *penA-*34-harboring ST-1901 isolates from Japan (two isolates from Aichi and one isolate from Gifu prefectures) isolated in February–March 2005 and newly sequenced in this study using MinION and MiniSeq. These three strains were isolated via surveillance by Gifu University, out of the 51 ST-1901 strains (95 strains across various STs) obtained in 2005 in total. In addition, an ST-1901 strain (12–032) isolated in 2000 in Kanagawa, Japan [11], harboring *penA*-5 and exhibiting cephalosporin susceptibility was re-sequenced using the MinION platform to examine conserved genomic regions between it and the strains harboring *penA*-34. Five other ST-1901 isolates dating from 2001 to 2004 in Kanagawa, Japan, were sequenced using the Illumina MiSeq platform. These newly sequenced genomes were combined with those of 1066 publicly available genomes from 14 studies spanning 35 countries: USA, Japan, Canada, UK, Spain, Brazil, Portugal, New Zealand, Hungary, Norway, Ireland, Greece, Sweden, Slovenia, Germany, Poland, Belarus, Denmark, Slovakia, Belgium, Australia, Austria, Italy, Netherlands, France, Cyprus, Latvia, Malta, Russia, Philippines, Bulgaria, Chile, India, Finland, and Estonia (ordered by frequency) [9, 11, 13–23] (see Additional file 2: Table S1 for details). The publicly available genomes were selected based on a tree constructed in our previous study [24] such that they formed a group at the core-genome level.

For MinION sequencing, a MagAttract HMW DNA Kit (Qiagen, Hilden, Germany) was used for isolation of high molecular weight genomic DNA of each isolate, and the Rapid Sequencing Kit (SQK-RAD004) and R9.4 flow cells were used. For MiSeq sequencing, the genomic DNA of each isolate was extracted using a MagNA Pure LC DNA isolation kit on a MagNA pure LC instrument (Roche Diagnostics GmbH, Mannheim, Germany), which was used for Nextera XT library construction and genome sequencing (300 bp paired-end) using an Illumina MiSeq Reagent Kit v3 (600-cycle).

For ST-7363, 103 strains that were isolated from 1996 and 2005 in Kanagawa were sequenced using the Illumina MiSeq platform. These data were combined with 88 publicly available genomes [11, 14] (Additional file 3: Table S2).

### Antimicrobial susceptibility testing

The minimum inhibitory concentrations (MICs) for 3GCs (ceftriaxone and cefixime) and fluoroquinolones (ciprofloxacin and levofloxacin) of the newly sequenced historical strains isolated in Kanagawa were determined using the agar dilution method [25] at the Kanagawa Prefectural Institute of Public Health and Gifu University (Additional file 2: Table S1 and Additional file 3: Table S2). MIC measurements were repeated for the following strains for which the genotypes and phenotypes were initially discordant, and MICs that better matched the genotypes were subsequently used: GCGS0938 (ceftriaxone) and GCGS0627 (ceftriaxone) in ST-1901 using the E test method (bioMérieux) and GU250 (cefixime, ceftriaxone), GU431 (cefixime, ceftriaxone), and GU478 (cefixime, ceftriaxone) in ST-7363 using the agar dilution method. To define susceptible/resistant phenotypes, the following MIC cutoffs were used according to the European Union Committee on Antimicrobial Susceptibility Testing (EUCAST) [26]: susceptibility ≤ 0.125 μg/mL and resistance > 0.125 μg/mL for ceftriaxone and cefixime and susceptibility ≤ 0.03 μg/mL and resistance ≥0.06 μg/mL for ciprofloxacin. For the strains where MICs of fluoroquinolones were measured only for levofloxacin by the Gifu University (names starting with “GU_”), the MIC cutoff of ciprofloxacin was used, as that of levofloxacin is not defined in EUCAST; a previous study has shown that MIC_50_, MIC_90_, and MIC range of ciprofloxacin and levofloxacin were similar for a set of 87 isolates [27].

### Bioinformatic analyses

After genome assembly, the number of contigs and N50 of each isolate are summarized in Additional file 2: Table S1 and Additional file 3: Table S2. All isolates used in this study can be found on the pubMLST.org/neisseria database [28] where MLST, NG MAST, and NG STAR STs were determined along with the core genome groups of the isolates as described previously [29]. MLST typing of 158 out of the 1075 strains showed that they were not ST-1901, but were included in our dataset regardless, as they formed a large cluster with other ST-1901 strains as a core-genome group.

The clonal phylogeny of ST-1901-associated and ST-7363-associated core-genome groups with branch lengths corrected to account for homologous recombination were inferred using the standard model of ClonalFrameML [30], followed by BactDating [31]. The presence or absence of 3GC resistance determinants (*penA* alleles) and fluoroquinolone resistance determinants (nonsynonymous substitutions in *gyrA* and *parC* described in a review [10] and also examined in our previous study [11]), as well as the MICs of the antimicrobial drugs for each strain, were illustrated as heat maps using Phandango [32]. For 3GC resistance determinants, the allele numbering defined in NG-STAR database [33] was assigned to each *penA* allele [“penA_allele (NG-STAR)” column in Additional file 2: Table S1 and Additional file 3: Table S2].

The recombination events that generated the mosaic *penA* alleles were investigated using a nucleotide sequence alignment of *penA* and a 5 kb region downstream. The alignment was prepared based on a BLASTn search against either (1) the genome sequences of ST-1901- and ST-7363-associated core-genome groups or (2) a custom database including 140 strains isolated in 1996–1997 in the Japanese prefecture of Kanagawa (Additional file 4: Table S3) and 204 strains isolated via genomic surveillance in 2015 in the Japanese prefectures of Kyoto and Osaka [11], both of which were not confined to ST-1901 or ST-7363, but included various STs. The alignment was manually examined using Jalview [34] to analyze nucleotide sequence identity and detect recombined fragments.

## Results

### ST-1901-associated core-genome group and resistance to 3GCs

**Fig. 1:**
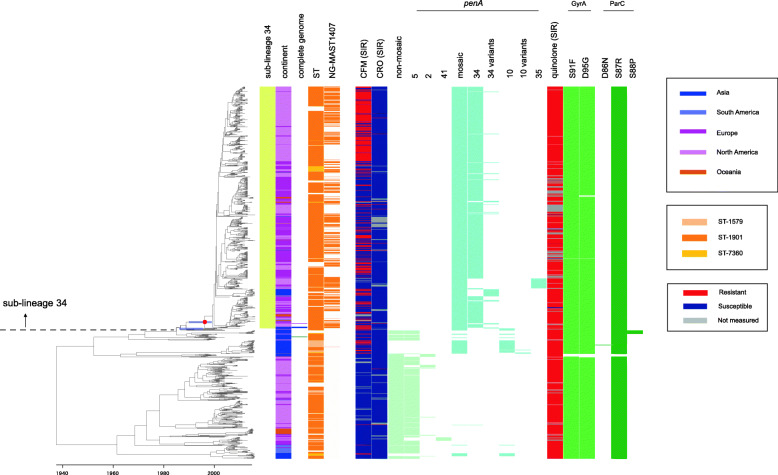
Whole genome sequence dated phylogeny, antimicrobial resistance, and genetic polymorphisms in the ST-1901-associated core-genome group. Left: A clonal dated phylogeny with corrected branch lengths to account for homologous recombination. In the heat map, the 3rd column titled “complete genome” shows the three Japanese strains harboring *penA*-34 from 2005 in blue and the ancestral Japanese strain (at the bottom) harboring *penA*-5 in green, whereas the WHO_Y (F89) strain is in pink. In the 4th column titled “ST”, ST-1901 and its single locus-variants are colored using different colors as shown in our previous study [11]. In the 6th and 7th column, susceptible/resistant (S/R) categories according to the EUCAST breakpoint of 3GCs (cefixime CFM and ceftriaxone CRO) are shown. The columns were colored gray when the MIC values were missing. In the 8th column, the presence (light yellow-green) or absence of any non-mosaic *penA* allele is shown. In the 9th–12th columns, the presence (light yellow-green) or absence of a specific non-mosaic *penA* allele is shown. The 13th column shows the presence (cyan) or absence any mosaic *penA* allele. The 14th–18th columns show presence (cyan) or absence a specific mosaic *penA* allele (specifically, 34 and its variants [35], 10 and its variants [35], and 35 [17]). The 19th column shows the susceptible/resistant (S/R) categories of fluoroquinolones (mostly ciprofloxacin, and much less frequently, levofloxacin) according to the EUCAST breakpoint. The 20th–21st columns show the presence (yellow-green) or absence of nonsynonymous amino acid changes compared to the wild type in GyrA. The 22nd–24th columns show the presence (green) or absence of nonsynonymous amino acid changes compared to the wild type in ParC. In the clonal dated phylogeny at the left, a red circle indicates emergence of the sub-lineage 34, whereas two purple lines indicate 95% confidence intervals examined in the main text (emergence time of the sub-lineage 34, and that of one of the three sub-lineages harboring *penA*-10)

A clonal dated phylogeny of ST-1901-associated core-genome group gonococci was inferred (Fig. 1) for 1075 isolates sampled from 35 countries dating from 1992 to 2016 (Additional file 2: Table S1). Identified core-genome groups corresponded to the recently designated “core-genome group cluster 3,” which had been identified using a locus threshold of 400 or fewer locus differences [29] (Additional file 2: Table S1), except for two isolates, which belonged to cgc_400 groups 18 and 221. Parameter estimates from ClonalFrameML were consistent with those observed previously [11] (Additional file 1). In general, isolates harboring the mosaic *penA* alleles (colored cyan in Fig. 1) and others were separated into two clusters, hereafter named “sub-lineage 34” and “susceptible sub-lineage,” respectively. The sub-lineage 34 accounts for the majority of isolates encoding mosaic *penA* alleles and harbors *penA-*34 and its variants (highlighted in the 1st column titled “sub-lineage 34” of Fig. 1 in yellow). This lineage was estimated to have emerged (red circle in the tree) between May 1990 and July 1999 (95% credibility interval). The sub-lineage 34 is shown as an enlargement in addition to its phylogenetic neighbor located at the bottom in Figure S1 (Additional file 5: Figure S1). As shown in the 2nd column headed “continent” in Fig. 1, most strains were isolated from North America (light pink, 50.6%) and Europe (pink, 42.6%) mainly through the Gonococcal Isolate Surveillance Project (GISP) [9] (accounting for 80.1% of the strains isolated from North America) and the European Gonococcal Antimicrobial Surveillance Programme (Euro-GASP) [13] (accounting for 64.3% of the strains isolated from Europe), respectively, while very few strains were isolated from Asia (blue, 3.7%), Oceania (brown, 2.3%), and South America (light blue, 0.9%). In the sub-lineage 34, strains with the oldest isolation date were from 2005, among which three were from Japan and two from the USA. Complete genome sequences of the three Japanese strains were phylogenetically located at the base of the sub-lineage 34 tree (highlighted in the 3rd column “complete genome” of Fig. 1 in blue). The three Japanese isolates (GU029, GU058, and GU092) were collected in the Japanese prefectures of Aichi and Gifu in February–March 2005, indicating that none of them were likely closely related to an ancestor of the strains that spread to other countries.

To provide an outgroup for sub-lineage 34, we obtained the complete genome sequence of an isolate, DRR129099 (12-032 in our previous study [11]), from the Japanese prefecture of Kanagawa in July 2000. This isolate harbors a non-mosaic *penA* allele and is phylogenetically close to the sub-lineage 34 (highlighted in green in the 3rd column headed “complete genome” of Fig. 1). Alignment of the complete genomes of the four isolates and the reference isolate WHO_Y (F89) included in the sub-lineage 34 (highlighted in pink in the 3rd column headed “complete genome” of Fig. 1) revealed conserved genomic regions among the reference isolate in the sub-lineage 34, DRR129099 harboring a non-mosaic *penA*-5 allele, and one of the isolates (GU092) encoding *penA*-34 (1st, 4th, and 5th genome in Additional file 5: Figure S2). This indicated that the overall genomic structure was maintained during evolution from the ancestor of the isolates in the sub-lineage 34 that spread to other countries.

In the whole ST-1901-associated core-genome group, ST-1901 and its single locus variants, ST7360 and ST1579, are colored in the “ST” column in Fig. 1. The 91 (8.5%) other strains comprised 21 STs. The top five STs (ST-9365, ST-10312, ST-8153, ST-10241, and ST-13840) accounted for 62.6% of the samples (Additional file 5: Figure S3) and belonged to the ST-1901-associated core-genome group, although the nucleotide sequences of the seven loci differed from those of ST-1901. Similarly, in the sub-lineage 34, 51.7% of the strains were NG-MAST1407 (colored in the “NG-MAST1407” column in Fig. 1), while the other strains were classified into 274 types or as undetermined according to the database of nucleotide sequences of the two highly variable NG-MAST loci (*porB* and *tbpB*).

Most isolates harboring the “non-mosaic” *penA*-5 were located in the susceptible sub-lineage, whereas other isolates harboring *penA*-5 were present immediately outside the sub-lineage 34, suggesting that *penA*-5 was ancestral and that *penA*-34 evolved from it (Fig. 1). The sub-lineage harboring *penA*-34 and its variants appeared to be monophyletic, whereas the entire phylogeny of ST-1901-associated core-genome group included three separate mosaic *penA*-10 allele acquisitions, one of which was observed in this study to be phylogenetically close to sub-lineage 34. This included the oldest isolates harboring *penA*-10 from 2000 to 2001 in Japan [14] (Additional file 5: Figure S1), which were not included in the previous study [11]. This suggests that *penA*-34 was generated via *penA*-10 from the ancestral *penA*-5 allele.

A small cluster of isolates encoding mosaic *penA*-35 was found in the sub-lineage 34, which consisted of 27 isolates that were isolated in India, Canada, UK, and USA from 2008 to 2011 and were susceptible to cefixime and ceftriaxone. A comparison of the amino acid sequences of *penA*-34 and 35 indicated that the differences in the amino acid residues across the sequence were likely consequences of HGT events spanning the entire *penA* locus from an unknown source outside the sub-lineage 34 (Additional file 5: Figure S4).

### Dated phylogeny of ST-7363-associated core-genome group, susceptibility to the 3GCs, and *penA* alleles

**Fig. 2:**
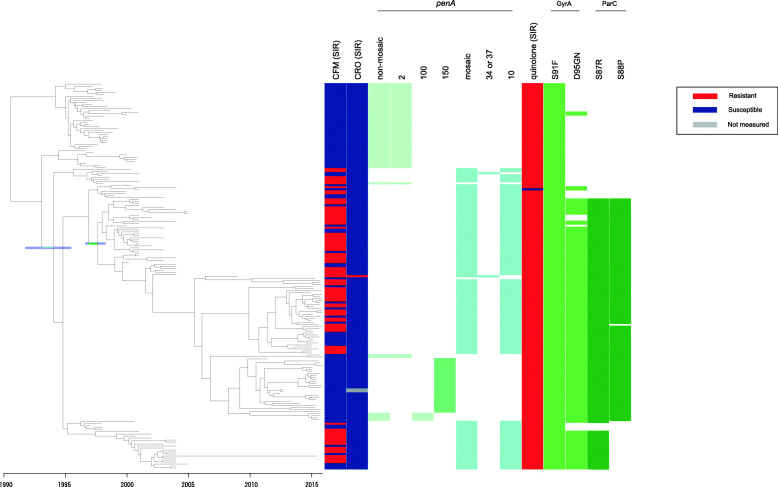
Whole genome sequence dated phylogeny, antimicrobial, and genetic polymorphisms in the ST-7363-associated core-genome group. The columns in the heat map are almost the same as those in Fig. 1, although the first five columns in Fig. 1 have been omitted here. The 1st and 2nd columns show the susceptible/resistant (S/R) categories according to the EUCAST breakpoint of cefixime (CFM) and ceftriaxone (CRO). The 3rd column shows the presence (light yellow-green) or absence of any non-mosaic *penA* allele. The 4th–6th columns show the presence (light yellow-green) or absence of a specific non-mosaic *penA* allele. The 7th column shows the presence (cyan) or absence of any mosaic *penA* allele. The 8th–9th columns show the presence (cyan) or absence a specific mosaic *penA* allele (specifically, 10, and 34 or 37 (H041-type) [7]). The 10th column shows the susceptible/resistant (S/R) categories of fluoroquinolones (mostly ciprofloxacin, and much less frequently, levofloxacin) according to the EUCAST breakpoint. The 11th–12th columns show the presence (yellow green) or absence of nonsynonymous amino acid changes compared to the wild type in GyrA. The 13th–14th columns show the presence (green) or absence of nonsynonymous amino acid changes compared to the wild type in ParC. In the clonal dated phylogeny at the left, the two branches of interest examined in the main text are colored cyan and green, with 95% confidence interval of the two evolutionary events (acquisition of *penA*-10 and simultaneous amino acid substitutions at GyrA 95 and ParC 87-88) colored purple

A clonal dated phylogeny of ST-7363-associated core-genome group, corresponding to recently designated “core-genome group cluster 8” [29] (Fig. 2), showed that the isolates were broadly separated into two clusters, corresponding to the presence or absence of a mosaic *penA* allele. In contrast to the ST-1901-associated core-genome group, we observed that the majority of the ancestral non-mosaic *penA* allele was *penA*-2, the nucleotide sequence of which is identical to that of *penA*-5 except for the region from nucleotides 1598-1752, and the 3′ terminus of the coding sequence (*penA-*2 was also referred to as “*penA*-5 variant” [11]). Consistent with the results of our previous report, the majority of the mosaic *penA* was *penA*-10, with the exception of isolate H041 which was resistant to ceftriaxone [7], and one isolate harboring *penA*-34 (ERS311596, isolated in 2001) [14]. The phylogeny showed that *penA*-10 in ST-7363 was generated from the ancestral *penA*-2 (Fig. 2) (95% credibility interval September 1991–May 1995). In agreement with a prior observation [11], we noted that *penA*-150 (designated in the NG-STAR database, Fig. 2), susceptible to 3GCs, appeared to be a product of the recombination between the mosaic *penA*-10 or 37 (H041-type) alleles and *penA*-5.

### Proposed origin of mosaic *penA*-34 and 10 alleles

**Fig. 3:**
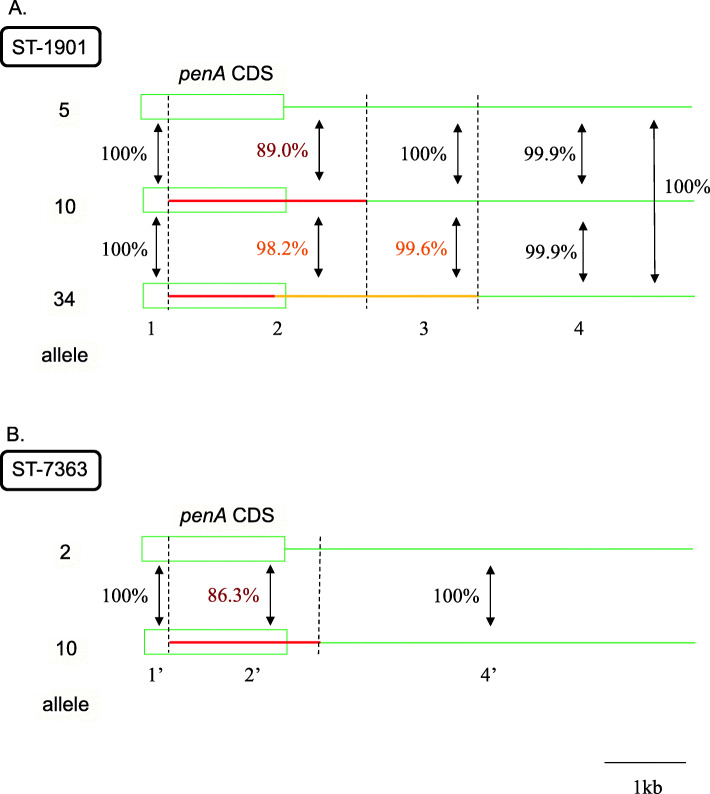
Schematic depiction of nucleotide sequence alignment of *penA* and its downstream sequence. **a** ST-1901-associated core-genome group. *penA*-5 (top), 10 (middle), and 34 (bottom). **b** ST-7363-associated core-genome group. *penA*-2 (top) and 10 (bottom). The coding sequence (CDS) of *penA* is shown as a rectangle. The recombined sequences in *penA*-10 and 34 are indicated by the red and orange lines, respectively

To further analyze the origins of mosaic *penA*-34 and *penA*-10, we compared their nucleotide sequences to those of the susceptible alleles in the ST-1901 and ST-7363-associated core-genome groups. Nucleotide sequence alignment of *penA*-5, 10, and 34 and their downstream sequences in ST-1901 is shown schematically in Fig. 3, and at the nucleotide level in Additional file 5: Figure S5. The 1st to 293rd nucleotides (region 1 in Fig. 3a) were identical, followed by the recombined mosaic region from 294th nucleotide (region 2, indicated by the red line in *penA*-10 in Fig. 3a, and between red arrows in Additional file 5: Figure S5), in which sequence identity between *penA*-5 and *penA*-10 with their downstream sequences was 89.0%, whereas sequence identity between *penA*-10 and *penA*-34 with their downstream sequences was 98.2%. The coding sequences (CDS) of *penA-*10 and 34 were identical, with the exception of 105 bp at the 3′ end, indicated by the left part of the orange line at the end of *penA*-34 in Fig. 3a. In the following region 3, the downstream sequences of *penA*-5 and *penA*-10 were identical, whereas those of *penA*-10 and 34 harbored four individual polymorphisms and 99.6% sequence identity. In the following region 4, the nucleotide sequences of *penA*-5, 10, and 34 exhibited ≥ 99.9% sequence identity. Based on the results of nucleotide sequence comparison, the origin of *penA*-10 was most parsimoniously explained by an approximately 2.4 kb recombination event from the start of mosaic region 2 in a *penA*-5 background. The source is unknown, as BLASTn search against the NCBI nr database did not yield any hit in other *Neisseria* species with high (> 95%) sequence identity.

**Fig. 4:**
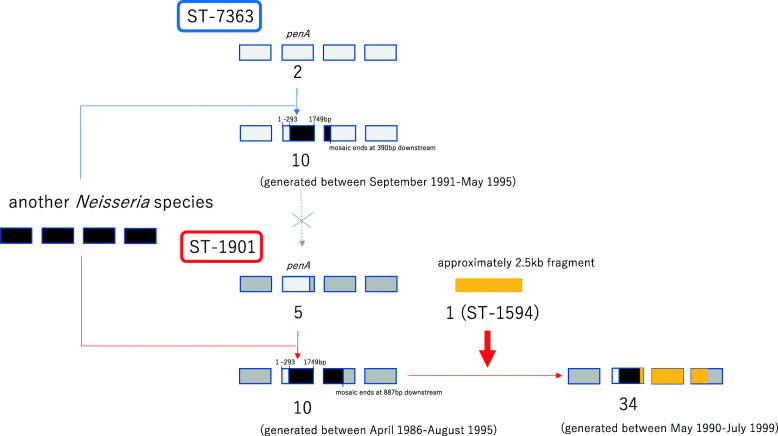
Schematic depiction summarizing the results regarding the origin of mosaic *penA*-10 and 34. Rectangles indicate genes, second of which is *penA*. In ST-7363 (top) and ST-1901 (bottom), the upper part shows the ancestral sequences (*penA*-2 and 5, respectively) while the lower part shows the recombined sequences

*penA* 34 and 10 differed in the 3′-terminal region, and the difference continued in their downstream nucleotide sequences in the regions 2 and 3. To investigate the possibility that *penA-*34 emerged via HGT in the background of *penA*-10, we aligned the nucleotide sequence corresponding to the orange line in regions 2 and 3 (approximately 2.5 kb, between orange arrows in Additional file 5: Figure S5) to the genome sequences of 140 strains isolated in 1996–1997 in the Japanese prefecture of Kanagawa (Additional file 4: Table S3, most of them newly sequenced in the present study) and the 204 Japanese strains isolated in 2015 [11], representing multiple STs. A maximum likelihood tree constructed from this alignment (Additional file 5: Figure S6) revealed that ST-1901 isolates encoding *penA*-34 and its variant were clustered with ST-1594 strains encoding *penA*-1. In particular, the nucleotide sequence was identical between *penA*-34 and *penA*-1, with the exception of a single polymorphism, whereas the *penA*-34 variant harbored a polymorphism at the C-terminus. The next most similar sequence was found in a strain (09-021) harboring *penA-*2 (located next to the ST-1594 strains encoding *penA*-1 in Additional file 5: Figure S7) and containing five polymorphisms compared to the sequence of *penA*-1. These results suggested that the origin of *penA*-34 can be explained by two recombination events: first, a recombination event from a commensal *Neisseria* into a *penA*-5 background, resulting in *penA*-10 (red line in *penA*-10 in Fig. 3a); second, a recombination event from *penA*-1 into the *penA*-10 background resulting in *penA*-34 (orange line in *penA*-34 in Fig. 3a and orange rectangle in Fig. 4).

Although no study has reported the prevalence of the *penA*-1 allele in Japan, analysis of genome sequence data of the 140 isolates in 1996–1997 and those of 204 isolates in 2015 collected via surveillance of symptomatic cases showed that the *penA*-1 allele was encoded only in ST-1594 and that it accounted for 3.6% and 7.8% of cases (the 7th and 5th most frequent ST) among the collected strains in 1996–1997 and 2015, respectively.

Similar to ST-1901, *penA*-2 and 10 of ST-7363 exhibited identical nucleotide sequence from nucleotide 1 to 293, followed by the mosaic region from nucleotide 294 to 390 bp downstream of the end of *penA* (region 2′, indicated by the red line in Fig. 3b and between red arrows in Additional file 5: Figure S6). This is most parsimoniously explained by a recombination event, although the source of this DNA segment is unknown (Fig. 4). Comparison of the length of the recombined regions between ST-1901 and ST-7363 indicated that *penA*-10 in ST-7363 was unlikely to be the origin of *penA*-10 in ST-1901, as the recombined fragment was shorter than that of ST-1901 (Fig. 4).

### Analysis of susceptibility to fluoroquinolones and examination of mutations in *gyrA* and *parC* in the dated phylogenies

For fluoroquinolone resistance, the dated phylogenies also revealed distinct evolutionary paths for the ST-1901- and ST-7363-associated core-genome groups. Almost all isolates in both core-genome groups were resistant to fluoroquinolones (minimal inhibitory concentration (MIC) > 0.06 μg/mL, using the EUCAST breakpoint) and harbored the GyrA 91F substitution. In the ST-1901-associated core-genome group, the substitutions at GyrA D95G and ParC S87R were shared among most isolates, including the clade encoding the non-mosaic *penA* (Fig. 1). The substitution at ParC S88P was found only in a small cluster of 11 strains (bottom; Fig. 1). In contrast, in the ST-7363-associated core-genome group, a dominant sub-lineage contained amino acid substitutions at GyrA D95GN, ParC S87R, and ParC S88P, which arose within a short time (highlighted as a green horizontal line in Fig. 2; 95% credibility interval August 1996–March 1998). These substitutions arose after the acquisition of *penA*-10, which is inferred to have occurred on a more ancestral node: the 95% credibility intervals for the dates of these two events do not overlap. The output of ClonalFrameML used for constructing the clonal phylogeny did not include a signature of the recombination importing the GyrA and ParC substitutions, suggesting that they arose via independent mutations.

## Discussion

An extensive analysis of *N. gonorrhoeae* ST-1901 and ST-7363-associated core-genome groups has improved the resolution of the time-resolved phylogenies of these important gonococci. Newly sequenced, historical Japanese isolates included three ST-1901 gonococci harboring the mosaic *penA*-34 (NG-MAST1407), which accounted for the majority of isolates with reduced susceptibility to 3GC in the USA and Europe. In the ST-1901-associated core-genome group, the three earliest isolates were obtained in Japan from February–March 2005, while two others originated in the USA (GCGS0944, GCGS0920) in 2005. Although we must consider the potential sample bias in the large dataset of ST-1901-associated core-genome group, in which 83% of the strains were isolated in Europe and North America, the time-resolved tree suggested that the three Japanese isolates were phylogenetically closest to the unsampled ancestor, consistent with the hypothesis that this sub-lineage likely originated in Japan [12].

The time-resolved trees of ST-1901 and ST-7363-associated core-genome groups enabled us to estimate the timing of the emergence of three genetic determinants of antimicrobial resistance: (i) mosaic *penA*-34 in ST-1901 (May 1990–July 1999), (ii) mosaic *penA*-10 in ST-1901 (April 1986–August 1995) and in ST-7363 (September 1991–May 1995), and (iii) GyrA D95GN and ParC S87R-S88P in ST-7363 (August 1996–March 1998).

How do these estimated dates correlate to antibiotic use? Although there were no national data on antibiotic usage at that time in Japan, a 1999 survey on the use of antimicrobial drugs in fourteen hospitals and four clinics mostly in a prefecture in Japan showed that 50% cases (nine out of eighteen) exclusively used fluoroquinolones for the treatment of urethritis (Dr. Mitsuru Yasuda, unpublished data). Similarly, another survey of clinics in Fukuoka in Japan in the late 1990s reported that fluoroquinolones were most frequently used (46%) as first-line treatment for gonorrhea and specifically, a 7-day course of levofloxacin (200 mg twice a day or 100 mg three times a day) was most frequently used in the treatment of gonococcal infections [36]. The regimen used in Japan in the late 1990s exceeded the single ciprofloxacin dose recommended in the USA, and may have contributed to selection of the *gyrA* and *parC* mutations, resulting in increased fluoroquinolone resistance.

A survey in Fukuoka, Japan, also showed that in the 1990s, fluoroquinolones were most frequently used (46%) as the first-line of treatment for gonorrhea, followed by penicillin with or without a β-lactamase inhibitor (28%), and cephems (16%) [36]. In 1999, the reported MIC_90_ for cefixime for clinical isolates of *N. gonorrhoeae* was 0.06 to 0.125 μg/mL, which indicated the presence of isolates with cefixime MIC = 0.125 μg/mL. This prompted the conclusion that a single dose of 400 mg cefixime used in the USA would not eradicate ≤ 95% of the clinical strains of *N. gonorrhoeae* [37]. The first Japanese STI treatment guidelines in 1999 described a regimen of six 100 mg doses of cefixime at a 12-h interval. Between 1999 and 2001, a regimen of 200 mg cefixime at 6 h interval was proposed, although it eradicated only 88.2% and 54.5% *N. gonorrhoeae* isolates with MIC_90_ and MIC values of 0.125 μg/mL, respectively [37]. These regimens and the observed circulating resistance might have contributed to the selection and dissemination of gonococcal strains with reduced susceptibility to cephalosporins. Although information regarding the alleles responsible for the cefixime MIC of 0.125 μg/mL at that time is lacking, the rising MICs and shifting patterns of antibiotic use reflect the dynamic interplay between the evolution of cefixime resistance and shifting treatment strategies.

Regarding the GyrA D95GN and ParC S88P amino acid substitutions in ST-7363, which are responsible for fluoroquinolone resistance, it is interesting to note that a sub-lineage harboring two substitutions (GyrA D95GN and ParC S87R, but not at ParC 88), has not been sampled since 2005 (except for the GU294 strain isolated in 2015 as indicated by the long branch at the bottom of Fig. 2). Although the overall sampling from 2005 to 2014 was insufficient, the clonal dated phylogeny shows that the sub-lineage was dominant around 2003 but became very rare in 2015, which suggests the importance of the substitution at ParC 88 or potentially another mutation that became dominant in ST-7363 by 2015. Alternatively, the stochasticity of transmission or some other factor (perhaps susceptibility to local treatments) might have led to the decrease in frequency of this sub-lineage.

We proposed two potential recombination events for the generation of *penA-*34 in ST-1901: recombination with another *Neisseria* species that generated *penA*-10, followed by another recombination event with a strain harboring *penA*-1 that converted *penA*-10 to *penA*-34. The lengths of the recombined sequence were approximately 2.3 kb and 2.5 kb, respectively, which is consistent with a recent estimate (2.5 kb) of the mean of the geometrically distributed DNA tract lengths transferred between donors and recipients in *N. gonorrhoeae* [38]. Similarly, recombination with a donor susceptible to 3GC was previously reported in *N. gonorrhoeae* in Japan [11] and the USA [9, 15], although it led to loss of the resistance phenotype. Of note, the inference of recombination is based on sampling, and we cannot rule out the possibility that recombination with a currently unsampled strain might have generated *penA*-34. Nevertheless, we can speculate that a strain harboring *penA*-1 is currently the most likely donor of the recombination that generated *penA*-34 from *penA-*10.

The reason behind the dominance of ST-1901 harboring *penA*-34, but not ST-7363 harboring the same allele, remains unsolved. In the ST-7363 group, *penA*-10 was dominant, and there was only one isolate with *penA*-34, identified in 2001. Data indicating significant difference in fitness between *penA*-10 and 34 in vitro are lacking. Possibly, the differences in fitness, selection, and dissemination between *penA*-10 and 34 depended on the genetic background of those sequence types and environmental factors at the time of selection.

Unfortunately, data regarding the potential environmental factors, such as national or regional information of antimicrobial use at the time of their emergence and dissemination, are inaccessible. Further studies are warranted to prospectively collect such data and isolates, and conduct integrative analyses of genome sequences, antimicrobial susceptibility, and the environmental factors to monitor, understand, and control emergence and dissemination of new resistance determinants of public health importance. A recent study that analyzed the genome and antimicrobial susceptibility data of *N. gonorrhoeae* isolates from 1928 to 2013 in Denmark [39] reported that no isolate was interpreted to be ciprofloxacin-resistant (MIC > 0.06 μg/mL) until the 1980s. The percentage of isolates resistant to ciprofloxacin increased from 0 to 14.3% in 1990s. Five isolates from the 1950–1970 contained a GyrA S91T amino acid substitution, although all of them were susceptible to ciprofloxacin. Compared to the recent study, the isolates analyzed in our study were all collected after 1990: the oldest strain of the ST-1901-associated core-genome group was collected in 1992 and that of ST-7363 in 1996. Further studies are warranted to conduct genome sequencing of historical Japanese strains collected before 1990s to identify isolates phenotypically susceptible to fluoroquinolones and explore when and how the individual amino acid substitutions of GyrA S91F, D95G, and ParC S87R in ST-1901 and that of GyrA S91F in ST-7363 may have occurred.

Our previous study revealed that there are different evolutionary pathways of the two major core-genome groups regarding the mosaic *penA* alleles responsible for resistance to 3GC [11]. The present study increased our understanding by elucidating the following major points: (1) *penA*-34 in ST-1901 was likely generated from *penA*-10 via two recombination events; (2) the *penA*-10 allele in the ST-1901-associated core-genome group emerged in at least three distinct Japanese sub-lineages independently, one of which was phylogenetically adjacent to the sub-lineage harboring *penA*-34; and, (3) the *penA*-10 allele in ST-7363 was unlikely to be a source of HGT that generated *penA*-10 in ST-1901-associated core-genome group, as the recombined fragment was shorter in ST-7363. Regarding the second and third points, although the source is not yet identified, the *penA*-10 allele was possibly generated via recombination with another commensal *Neisseria* species, similar to another mosaic *penA*-60 allele (FC428-type [40]) that was likely generated via recombination with *Neisseria cinerea* [41]. The single acquisition of *penA*-10 in ST-7363 was revealed in both the previous and present studies, although our understanding regarding its relationship with *penA*-10 and *penA*-34 in the ST-1901-associated core-genome group, in terms of order of their generation, and possibility of recombination between the two core-genome groups, were improved in the present study. Furthermore, the present study demonstrated another interesting difference in fluoroquinolone resistance between the two core-genome groups since 1990s. ST-7363 was originally susceptible to 3GC and harbored an amino acid substitution only in GyrA 91 in the 1990s; the simultaneous amino acid substitutions at GyrA 95 and ParC 87 occurred in the sub-lineage in the short time after it acquired the mosaic *penA*-10, whereas in the ST-1901-associated core-genome group, all the substitutions in GyrA 91, 95, and ParC 87 were already observed in 1990s in most strains (99.2%, 1066/1075), including the sub-lineage mostly susceptible to 3GC (bottom; Fig. 1).

## Conclusions

In summary, after combining the previously published dataset from 35 countries with the new genome sequence data and the antimicrobial susceptibility data of historical gonococcal isolates from Japan, we described the possible pathways of emergence of cephalosporin-resistant genes and fluoroquinolone-resistant mutations of two globally circulating *N. gonorrhoeae* core-genome groups. We further discussed the dynamic interplay between the evolution of antibiotic resistance and treatment regimens during time period of the emergence of genetic determinants of antimicrobial resistance. Such elucidation of evolutionary pathways will be useful for understanding and controlling the current and future evolution and spread of the pathogen and resistance determinants driven by recombination and selective pressure of antibiotic use.

## Supplementary Information

**Additional file 1.** Supplementary Methods and Results.

**Additional file 2: Table S1.** List of isolates in the ST-1901-associated core-genome group (according to the order in Fig. 1).

**Additional file 3: Table S2.** List of isolates in the Japanese ST-7363-associated core-genome group (according to the order in Fig. 2).

**Additional file 4: Table S3.** List of Japanese isolates sampled from 1996 to 1997.

**Additional file 5: Figure S1.** Whole-genome sequence, dated phylogeny, resistance patterns of the antimicrobials, and genetic polymorphisms in the ST-1901-associated sub-lineage carrying *penA*-34. **Figure S2.** Whole-genome alignment of the ancestral strain encoding *penA*-5, three strains encoding *penA*-34 dating from 2005, and the reference WHO_Y (F89) strain encoding *penA*-34. **Figure S3.** Frequency distribution of other 21 STs in the ST-1901-associated lineage. **Figure S4.** Amino acid sequence alignment of *penA*-34 and 35. **Figure S5.** Nucleotide sequence alignment of *penA* and its downstream sequences in the ST-1901-associated lineage. **Figure S6** Nucleotide sequence alignment of *penA* and its downstream sequences in the ST-7363-associated lineage. **Figure S7.** Maximum-likelihood tree of the recombined region (orange in Fig. 3).

## Data Availability

Metadata for each isolate, such as MLST, MIC, and genetic polymorphisms, are summarized in Additional file 2: Table S1 for the ST-1901-associated core-genome group and in Additional file 3: Table S2 for the ST-7363-associated core-genome group. All isolates used in this study can be found on the pubMLST.org/neisseria database (https://pubMLST.org/neisseria) [28]. The raw read data of the newly sequenced strains in Additional file 2: Table S1 and Additional file 3: Table S2, and those in Additional file 4: Table S3 are deposited at DDBJ (accession numbers DRA010497 and DRA010848, https://ddbj.nig.ac.jp/DRASearch/study?acc=DRP006595 and https://ddbj.nig.ac.jp/DRASearch/study?acc=DRP006590) and NCBI BioProject accession numbers PRJDB10182 (https://www.ncbi.nlm.nih.gov/bioproject/?term=PRJDB10182) [42] and PRJDB10572 (https://www.ncbi.nlm.nih.gov/bioproject/?term=PRJDB10572) [43].
