# Supplementary material for: Emergence and evolution of antimicrobial resistance genes and mutations in Neisseria gonorrhoeae
Source: Genome Med. 2021 Mar 30;13:51. doi: 10.1186/s13073-021-00860-8 (PMC8008663; doi:10.1186/s13073-021-00860-8)
Supplement: Supplementary file 1 — Additional file 1. Supplementary Methods and Results. [file 13073_2021_860_MOESM1_ESM.docx]

**Supplementary Methods**

**Genome assembly and annotation**

The Illumina read data (300 bp paired-end reads) of each isolate sequenced using MiSeq were used for *de novo* assembly using SPAdes [1], or Shovill [2]. The assembled contigs were checked to ensure that the coverage was at least 10× and the genome size was approximately equal to that of the type strain *N. gonorrhoeae* FA1090 (2.0−2.3 Mbp) according to our previous study [3]. The MinION read data of each of the four isolates of ST-1901 were used for *de novo* assembly using Canu version 1.7 [4]. Errors were corrected using three runs of Pilon for each contig assembled from the MinION reads [5] based on mapping of Illumina reads to the contigs using bowtie2 [6] with “very-sensitive” option. This step was followed by automated circularization of the contigs using Circlator [7] and adjustment of the 1^st^ position of the genome sequence to the start of *dnaA* using an in-house script. Each genome was annotated using Prokka [8].

**Construction of the clonal time-resolved phylogeny of ST-1901-associated and ST-7363-associated core-genome groups**

As in previous studies [9, 10], pairwise genome alignment between the reference genome and one of the other strains was performed using progressiveMauve [11] for SNP calling and evolutionary analyses in ST-1901 and ST-7363, which enabled the construction of positional homology alignments even for genomes with variable gene content and rearrangement. The complete genome sequences of the “WHO_Y” (F89) and “WHO_X” (H041) strains [12] were used as ST-1901 and ST-7363 references, respectively. The alignments were then combined into a multiple whole genome alignment, in which each position corresponded to that of the reference genome. This approach was validated [10] by comparing with another whole genome alignment using a pipeline [13] that similarly conducts pairwise genome alignment between a reference genome and one of the other genomes using MUMmer [14]. A maximum likelihood tree was constructed using PhyML [15] from the genome alignment containing the 10,844 core SNPs in ST-7363. A maximum likelihood tree was generated from the genome alignment containing the 28,306 core SNPs in ST-1901 using RAxML [16], as PhyML failed to complete the tree generating process. The default setting was used for RAxML, whereas for PhyML, we used the -m GTR -c 4 -a e parameters that indicate the GTR + G4 model of DNA substitution with estimation of the shape parameter of the gamma distribution by maximizing the likelihood. Using this as a starting tree, a clonal phylogeny was constructed in which possible homologous recombination regions were identified and branch lengths were corrected to account for homologous recombination using the standard model of ClonalFrameML [17]. The alignment sites that were present in at least 70% isolates were used to focus on core and fairly conserved sites with missing frequency ≤ 30%, as validated in our previous study [10]. TempEst [18] was used to root the phylogeny by minimizing the sum of the squared residuals from the regression of root-to-tips on sampling year. BactDating [19] was used, in which the significance of the temporal signal in both ST-7363 and ST-1901-associated core-genome groups was confirmed (the date randomization test, *P* < 10^-4^). BactDating was then run for 10 million Markov chain Monte Caro (MCMC) iterations. After completion, the effective sample size (ESS) of all parameters were confirmed as exceeding 400 and trace plots were confirmed as demonstrating well-mixed chains. A time-resolved phylogeny and 95% confidence interval for each branch in the tree was exported.

**Analysis of genetic AMR determinants**

For 3GC resistance determinants, the *penA* alleles were extracted based on a BLASTn search of the nucleotide sequence of mosaic *penA* alleles-10 and-34 and that of non-mosaic *penA*-5 (https://figshare.com/articles/penA_nucleotide_sequence_alignment_penA_5_X_XXXIV_and_1_/12203858) against each of the other genomes. When the mosaic *penA* alleles extracted based on the BLASTn search were truncated, sequence reads were mapped to the nucleotide sequence of *penA* alleles-10 and-34. Figure 1 shows the grouping of “*penA*-34 variants” for alleles that were 100% aligned to *penA*-34 and showed sequence identity of < 100% and > 99.5%, which was higher than that observed with *penA*-10. The “*penA*-10 variants” were grouped similarly.

For fluoroquinolone resistance determinants, nonsynonymous substitutions in specific regions of *gyrA* and *parC*, namely, amino acid positions 91 and 95 in GyrA, position 87 in ParC, and less frequently, positions 86 and 88 in ParC were examined. The presence or absence of the substitutions was detected using PointFinder [20].

**Inference of recombination events that generated mosaic *penA*-10 and 34 alleles**

A nucleotide sequence alignment of *penA* and a 5 kb region downstream of its 3′ end was prepared based on a BLASTn search against the genome sequences of ST-1901- and ST-7363-associated core-genome groups. The nucleotide sequences of *penA*-34 and its downstream region were used as query sequences in the BLASTn search. The nucleotide sequences obtained from the BLASTn were aligned using MAFFT v7.245 [21] and manually examined using Jalview [22] to analyze nucleotide sequence identity and detect recombined fragments for *penA*-10 and 34 in ST-1901 and *penA*-10 in ST-7363. The BLASTn search was also performed against the custom database of genome sequences of Japanese *N. gonorrhoeae* strains that were not confined to ST-1901 or ST-7363, but included various STs. The nucleotide sequences obtained using BLASTn were aligned using MAFFT v7.245 [21] and manually examined using Jalview [22], using which an alignment of the potentially recombined fragment was extracted. A maximum likelihood tree of the fragment was constructed using PhyML [15], which was mid-point rooted and visualized using FigTree version 1.4.3. To investigate the potential sources of the recombination, a BLASTn search was performed for each recombined fragment in the NCBI nucleotide database or the custom database of genome sequences of Japanese *N. gonorrhoeae* strains.

**Supplementary Results**

The ratio of rates of recombination and mutation in ST-1901 was estimated to be 0.64, whereas the ratio of the number of substitutions predicted to have been imported through recombination and point mutation was 5.96, a relatively high value among bacterial species (Vos and Didelot, 2009, *ISME*) and consistent with estimates at species level in our previous study (Yahara et al, 2018, *Microbial Genomics*). The average genetic distance between the donor and the recipient in recombination events was estimated to be 0.086 in ST-1901.

1. Bankevich A, Nurk S, Antipov D, Gurevich AA, Dvorkin M, Kulikov AS, Lesin VM, Nikolenko SI, Pham S, Prjibelski AD *et al*: **SPAdes: a new genome assembly algorithm and its applications to single-cell sequencing**. *J Comput Biol* 2012, **19**(5):455-477.

2. Seemann T. **Shovill: faster SPAdes assembly of Illumina reads**.[<https://github.com/tseemann/shovill>] (2018) (Accessed: December 29, 2020)

3. Ma KC, Mortimer TD, Hicks AL, Wheeler NE, Sanchez-Buso L, Golparian D, Taiaroa G, Rubin DHF, Wang Y, Williamson DA *et al*: **Adaptation to the cervical environment is associated with increased antibiotic susceptibility in Neisseria gonorrhoeae**. *Nat Commun* 2020, **11**(1):4126.

4. Koren S, Walenz BP, Berlin K, Miller JR, Bergman NH, Phillippy AM: **Canu: scalable and accurate long-read assembly via adaptive k-mer weighting and repeat separation**. *Genome Res* 2017, **27**(5):722-736.

5. Walker BJ, Abeel T, Shea T, Priest M, Abouelliel A, Sakthikumar S, Cuomo CA, Zeng Q, Wortman J, Young SK *et al*: **Pilon: an integrated tool for comprehensive microbial variant detection and genome assembly improvement**. *PLoS One* 2014, **9**(11):e112963.

6. Langmead B, Salzberg SL: **Fast gapped-read alignment with Bowtie 2**. *Nat Methods* 2012, **9**(4):357-359.

7. Hunt M, Silva ND, Otto TD, Parkhill J, Keane JA, Harris SR: **Circlator: automated circularization of genome assemblies using long sequencing reads**. *Genome Biol* 2015, **16**:294.

8. Seemann T: **Prokka: rapid prokaryotic genome annotation**. *Bioinformatics* 2014, **30**(14):2068-2069.

9. Zhang G, Leclercq SO, Tian J, Wang C, Yahara K, Ai G, Liu S, Feng J: **A new subclass of intrinsic aminoglycoside nucleotidyltransferases, ANT(3")-II, is horizontally transferred among Acinetobacter spp. by homologous recombination**. *PLoS Genet* 2017, **13**(2):e1006602.

10. Yahara K, Nakayama SI, Shimuta K, Lee KI, Morita M, Kawahata T, Kuroki T, Watanabe Y, Ohya H, Yasuda M *et al*: **Genomic surveillance of Neisseria gonorrhoeae to investigate the distribution and evolution of antimicrobial-resistance determinants and lineages**. *Microb Genom* 2018, **4**(8).

11. Darling AE, Mau B, Perna NT: **progressiveMauve: multiple genome alignment with gene gain, loss and rearrangement**. *PLoS One* 2010, **5**(6):e11147.

12. Unemo M, Golparian D, Sanchez-Buso L, Grad Y, Jacobsson S, Ohnishi M, Lahra MM, Limnios A, Sikora AE, Wi T *et al*: **The novel 2016 WHO *Neisseria gonorrhoeae* reference strains for global quality assurance of laboratory investigations: phenotypic, genetic and reference genome characterization**. *J Antimicrob Chemother* 2016, **71**(11):3096-3108.

13. Didelot X, Pang B, Zhou Z, McCann A, Ni P, Li D, Achtman M, Kan B: **The role of China in the global spread of the current cholera pandemic**. *PLoS Genet* 2015, **11**(3):e1005072.

14. Kurtz S, Phillippy A, Delcher AL, Smoot M, Shumway M, Antonescu C, Salzberg SL: **Versatile and open software for comparing large genomes**. *Genome Biol* 2004, **5**(2):R12.

15. Guindon S, Dufayard JF, Lefort V, Anisimova M, Hordijk W, Gascuel O: **New algorithms and methods to estimate maximum-likelihood phylogenies: assessing the performance of PhyML 3.0**. *Syst Biol* 2010, **59**(3):307-321.

16. Stamatakis A: **RAxML version 8: a tool for phylogenetic analysis and post-analysis of large phylogenies**. *Bioinformatics* 2014, **30**(9):1312-1313.

17. Didelot X, Wilson DJ: **ClonalFrameML: efficient inference of recombination in whole bacterial genomes**. *PLoS Comput Biol* 2015, **11**(2):e1004041.

18. Rambaut A, Lam TT, Carvalho LM, Pybus OG: **Exploring the temporal structure of heterochronous sequences using TempEst (formerly Path-O-Gen)**. *Virus Evolution,* 2016, **2**(1):vew007.

19. Didelot X, Croucher NJ, Bentley SD, Harris SR, Wilson DJ: **Bayesian inference of ancestral dates on bacterial phylogenetic trees**. *Nucleic Acids Res* 2018, **46**(22):e134.

20. Zankari E, Allesoe R, Joensen KG, Cavaco LM, Lund O, Aarestrup FM: **PointFinder: a novel web tool for WGS-based detection of antimicrobial resistance associated with chromosomal point mutations in bacterial pathogens**. *J Antimicrob Chemother* 2017, **72**(10):2764-2768.

21. Katoh K, Standley DM: **MAFFT multiple sequence alignment software version 7: improvements in performance and usability**. *Mol Biol Evol* 2013, **30**(4):772-780.

22. Waterhouse AM, Procter JB, Martin DM, Clamp M, Barton GJ: **Jalview Version 2--a multiple sequence alignment editor and analysis workbench**. *Bioinformatics* 2009, **25**(9):1189-1191.
