# Supplementary material for: Emergence and evolution of antimicrobial resistance genes and mutations in Neisseria gonorrhoeae
Source: Genome Med. 2021 Mar 30;13:51. doi: 10.1186/s13073-021-00860-8 (PMC8008663; doi:10.1186/s13073-021-00860-8)
Supplement: Supplementary file 5 — Additional file 5: Figure S1. Whole-genome sequence, dated phylogeny, resistance patterns of the antimicrobials, and genetic polymorphisms in the ST-1901-associated sub-lineage carrying penA-34. Figure S2. Whole-genome alignment of the ancestral strain encoding penA-5, three strains encoding penA-34 dating from 2005, and the reference WHO_Y (F89) strain encoding penA-34. Figure S3. Frequency distribution of other 21 STs in the ST-1901-associated lineage. Figure S4. Amino acid sequence alignment of penA-34 and 35. Figure S5. Nucleotide sequence alignment of penA and its downstream sequences in the ST-1901-associated lineage. Figure S6 Nucleotide sequence alignment of penA and its downstream sequences in the ST-7363-associated lineage. Figure S7. Maximum-likelihood tree of the recombined region (orange in Fig. 3). [file 13073_2021_860_MOESM5_ESM.pdf]

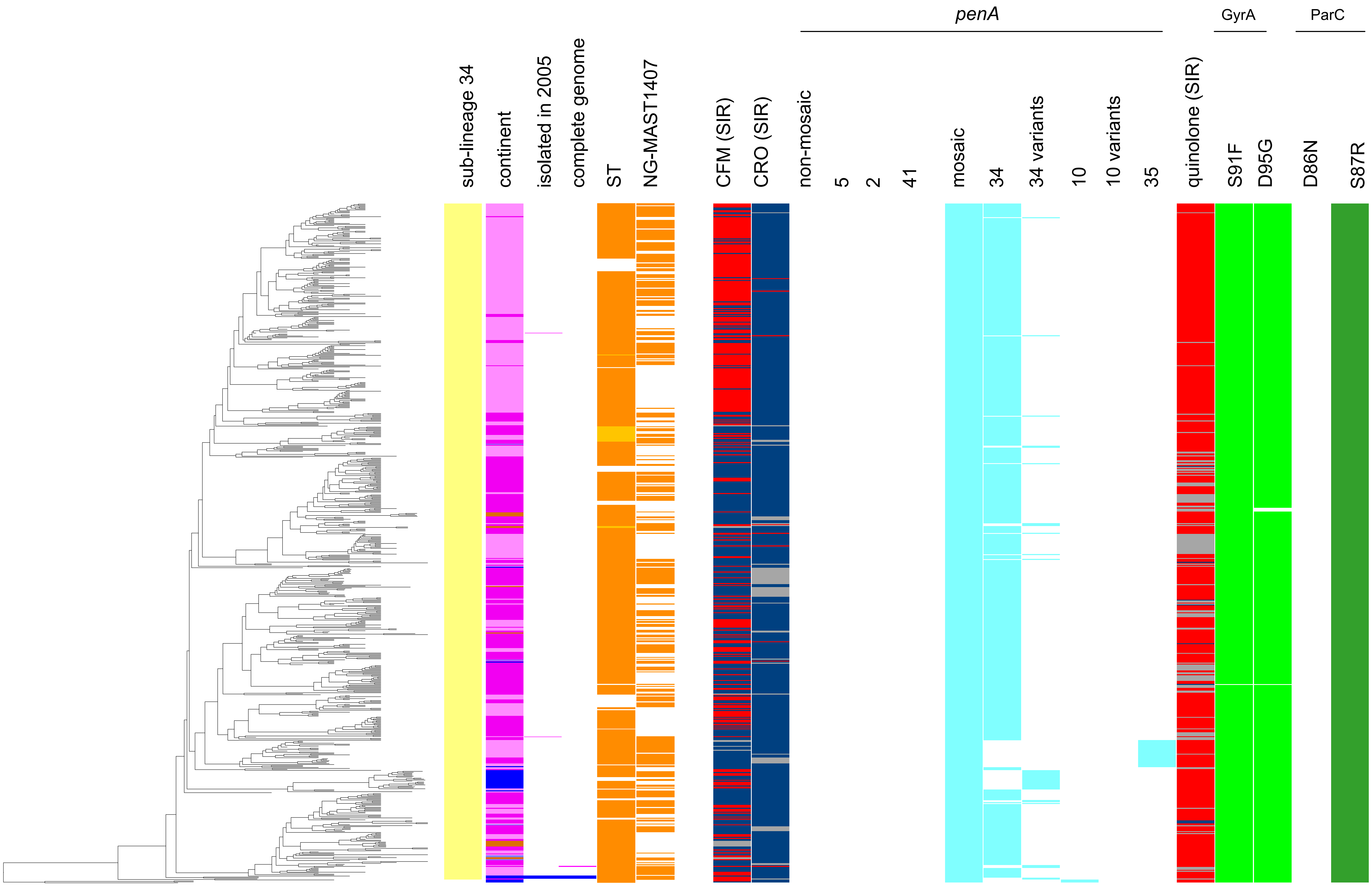

Fig S1. Whole-genome sequence dated phylogeny, resistance patterns of the antimicrobials, and genetic polymorphisms in the ST-1901-associated sub-lineage carrying *penA*-34. The columns in the heatmap are the same those in Fig. 1, except for addition of the 3rd column "isolated in 2005".

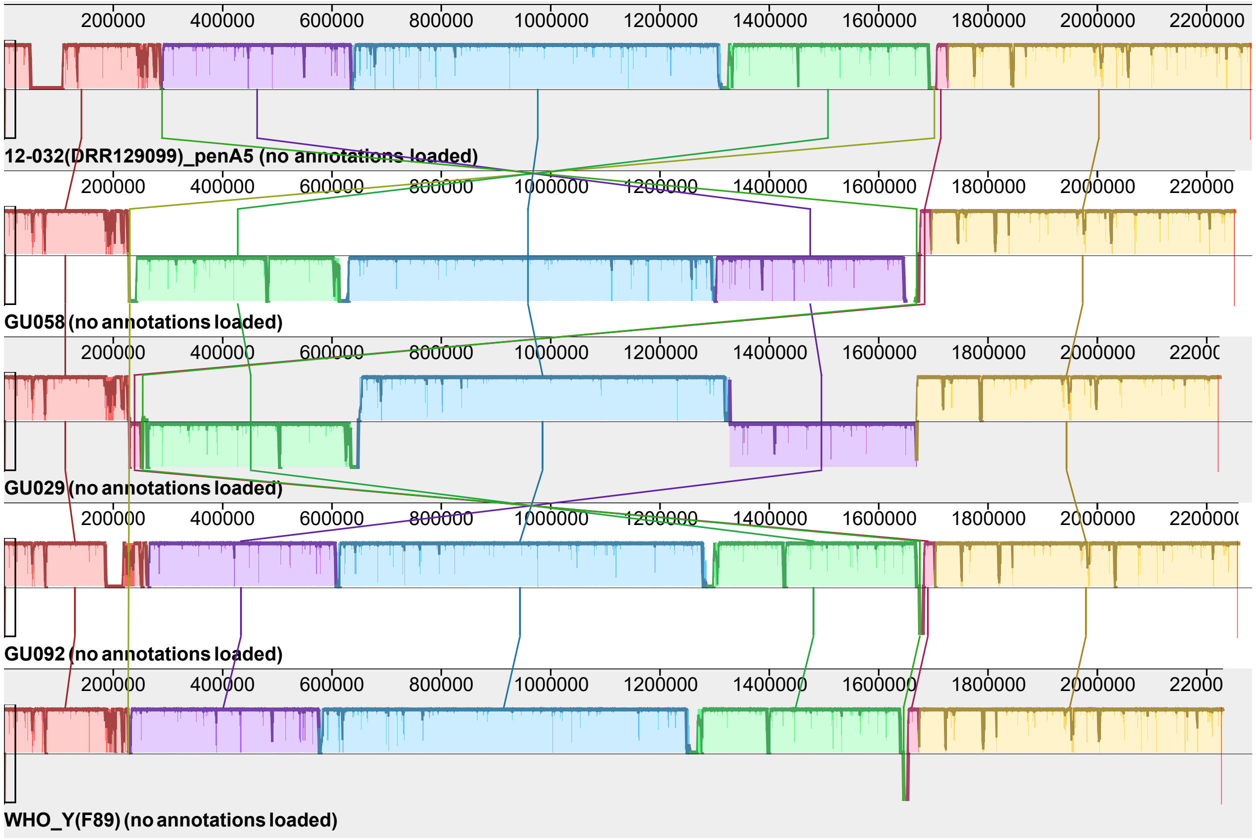

Fig S2. Whole-genome genome alignment of the ancestral strain encoding *penA*-5, three strains encoding *penA*-34 dating from 2005, and the reference WHO_Y (F89) strain encoding *penA*-34.

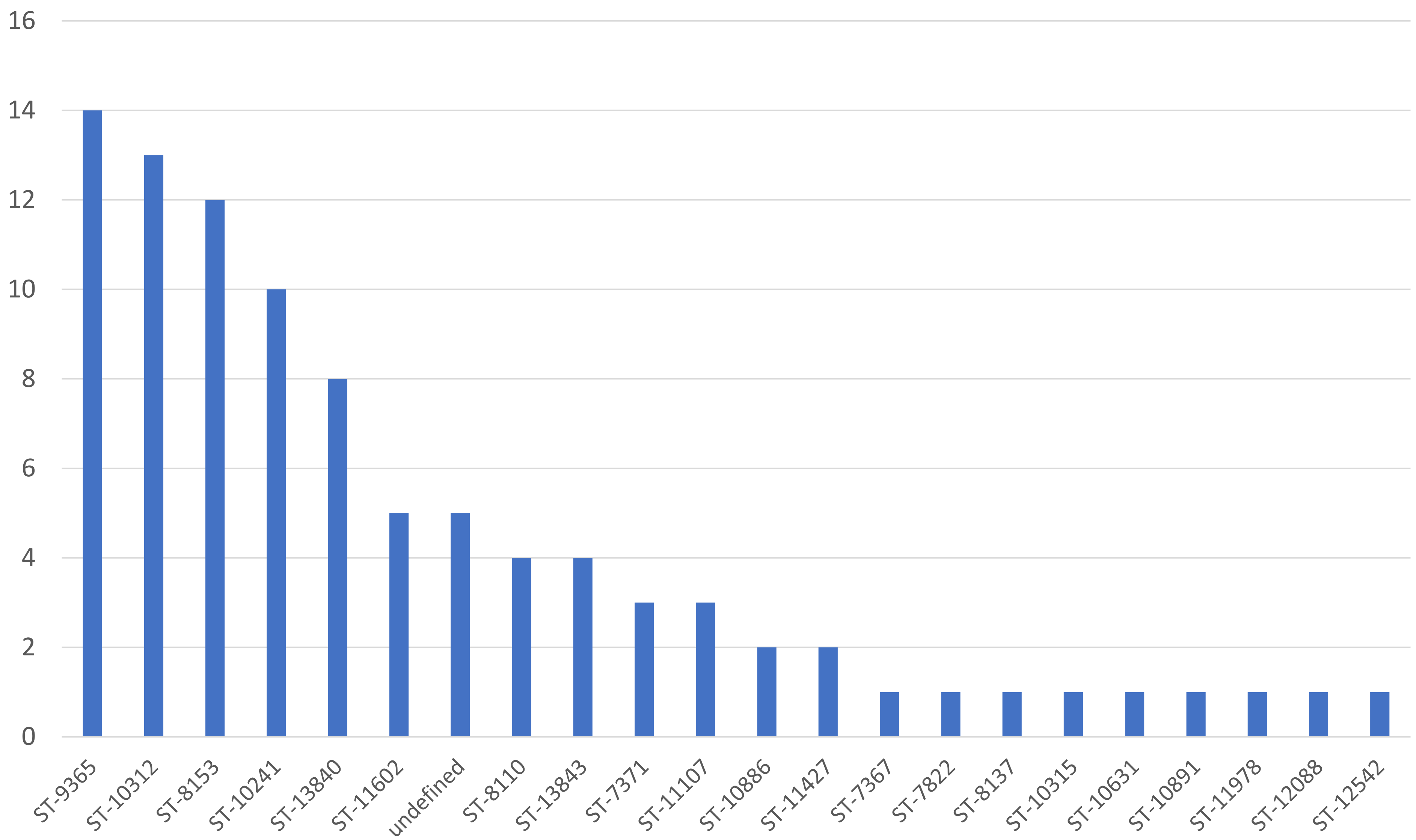

Fig S3. Frequency distribution of other 21 STs in the ST-1901-associated lineage. The columns in the heatmap are almost the same those in Fig. 1, although the first 5 columns in Fig. 1 are omitted.

```
penA_34    1 MLIKSEYKPRMLPKEEQVKKPMTSNGRISFVLMAMAVLFACL  42
penA_35    1 MLIKSEYKPRMLPKEEQVKKPMTSNGRISFVLMAIAVLFAGL  42

          43 IARGLYLQTVTYNFLKEQGDNRIVRTQALPATRGTVSDRNGA  84
          43 IARGLYLQTVTYNFLKEQGDNRIVRTQTLPATRGTVSDRNGA  84

          85 VLALSAPTESLFAVPKEMKEMPSAAQLERLSELVDVPVDVLR 126
          85 VLALSAPTESLFAVPKEMKEMPSAAQLERLSELVDVPVDVLR 126

         127 NKLEQKGKSFIWIKRQLDPKVAEEVKALGLENFAFEKELKRH 168
         127 NKLEQKGKSFIWIKRQLDPKVAEEVKALGLENFVFEKELKRH 168

         169 YPMGSLFAHVIGFTDIDGKGQEGLELSLEDSLHAGEGAEVVL 210
         169 YPMGNLFAHVIGFTDIDGKGQEGLELSLEDSLHAGEGAEVVL 210

         211 RDREGNIVDSLDSPRNKAPQNGKDIILSLDQRIQTLAYEELN 252
         211 RDRQGNIVDSLDSPRNKAPKNGKDIILSLDQRIQTLAYEELN 252

         253 KAVEYHQAKAGTVVVLDARTGEILALVNTPAYEPNKPGQADS 294
         253 KAVEYHQAKAGTVVVLDARTGEILALANTPAYDPNRPGRADS 294

         295 EQRRNRAVTDMIEPGSAMKPFTIAKALDSGKVDATDTFNTLP 336
         295 EQRRNRAVTDMIEPGSAMKPFTIAKALDSGKVDATDTFNTLP 336

         337 YKIGSATVQDTHVYPTLDVRGIMQKSSNVGTSKLSAMFTPKE 378
         337 YKIGPATVQDTHVYPTLDVRGIMQKSSNVGTSKLSAMFTPKE 378

         379 MYDFYHDLGVGVRMHSGFPGETAGLLRSWRRWQKIEQATMSF 420
         379 MYDFYHDLGVGVRMHSGFPGETAGLLRNWRRWRPIEQATMSF 420

         421 GYGLQLSLLQLARAYTVLTHDGELLPVSFEKQAVAPKGKRVI 462
         421 GYGLQLSLLQLARAYTALTHDGVLLPVSFEKQAVAPQGKRIF 462

         463 KASTAKKVRELMVSVTEAGGTGTAGAVDGFDVGAKTGTARKL 504
         463 KESTAREVRNLMVSVTEPGGTGTAGAVDGFDVGAKTGTARKF 504

         505 VNGRYVDYKHVATFIGFAPAKNPRVIVAVTIDEPTANGYYSG 546
         505 VNGRYADNKHIATFIGFAPAKNPRVIVAVTIDEPTAHGYYGG 546

         547 VVAGPPFKKIMGGSLNILGISPTKPLTAAAVKTPS-       581
         547 VVAGPPFKKIMGGSLNILGISPTKPLTAAAVKTPS*       582
```

Fig S4. Amino acid sequence alignment of *penA*-34 and 35.

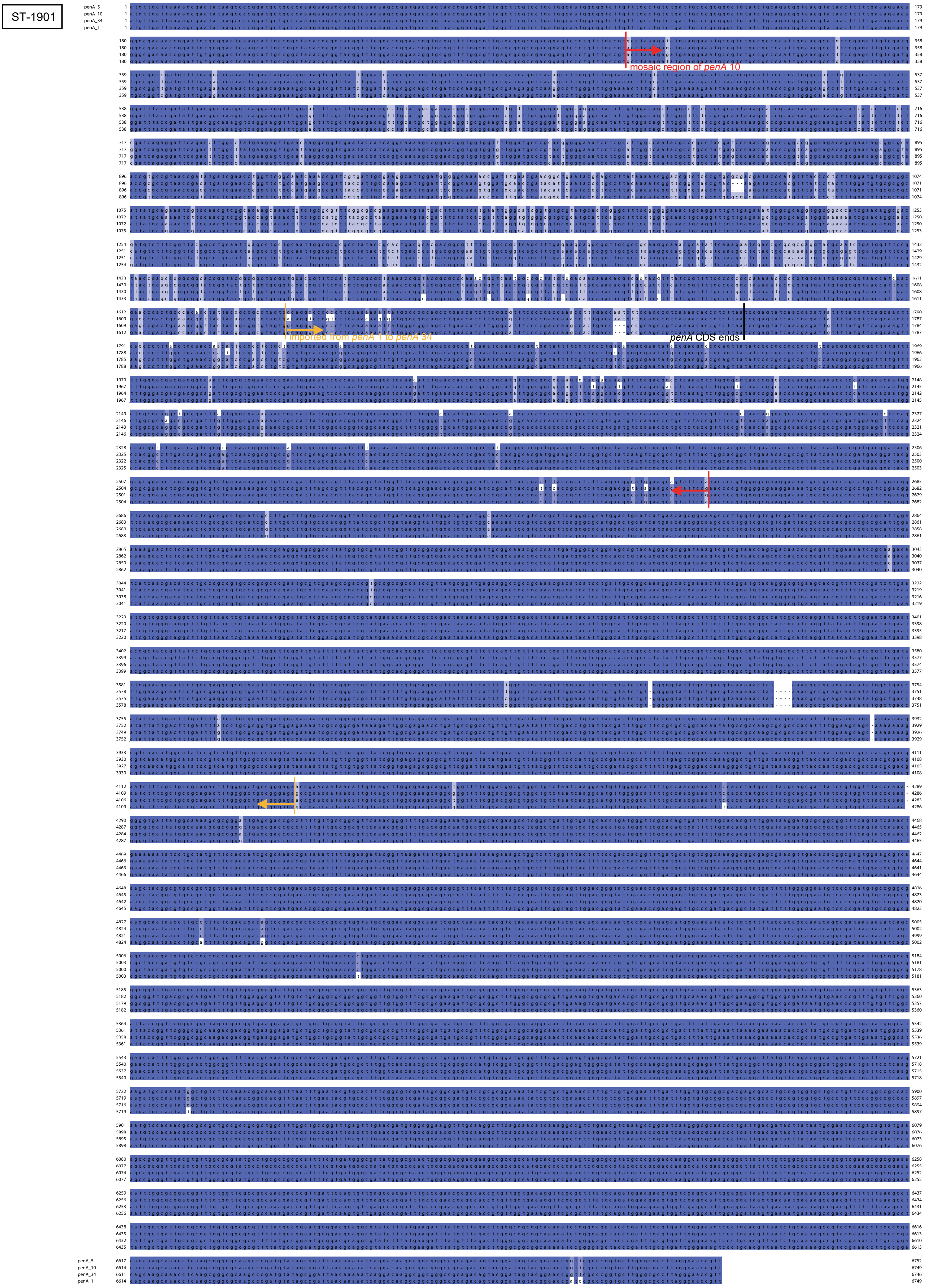

Fig S5. Nucleotide sequence alignment of *penA* and its downstream in the ST-1901-associated lineage. The four representative sequences of *penA*-5 (1st), 10 (2nd), 34 (3rd), and 1 (4th) and their downstream are shown. The two vertical red lines indicate the start and end of the mosaic region in *penA*-10 and its downstream, and the black vertical line indicates the end of *penA*. The orange arrows indicate the start and end of recombined region of *penA*-34 likely imported from *penA*-1 to *penA*-10.

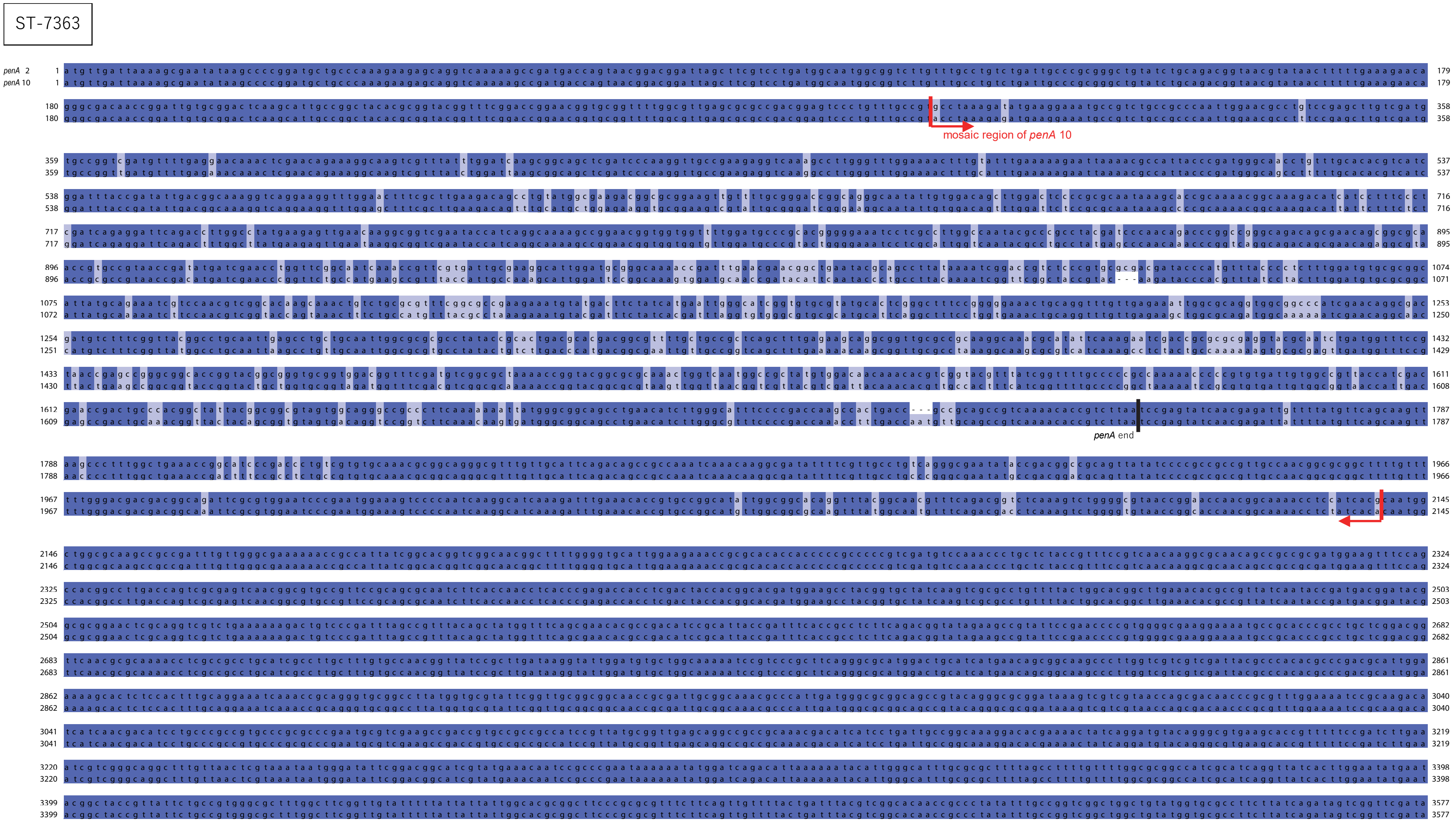

Fig S6. Nucleotide sequence alignment of *penA* and its downstream in the ST-7363-associated lineage. The two representative sequences of *penA*-2 (top) and 10 (bottom) and their downstream are shown. The two vertical red lines indicate the start and end of the mosaic region in *penA*-10 and its downstream, and the black vertical line indicates the end of *penA*.

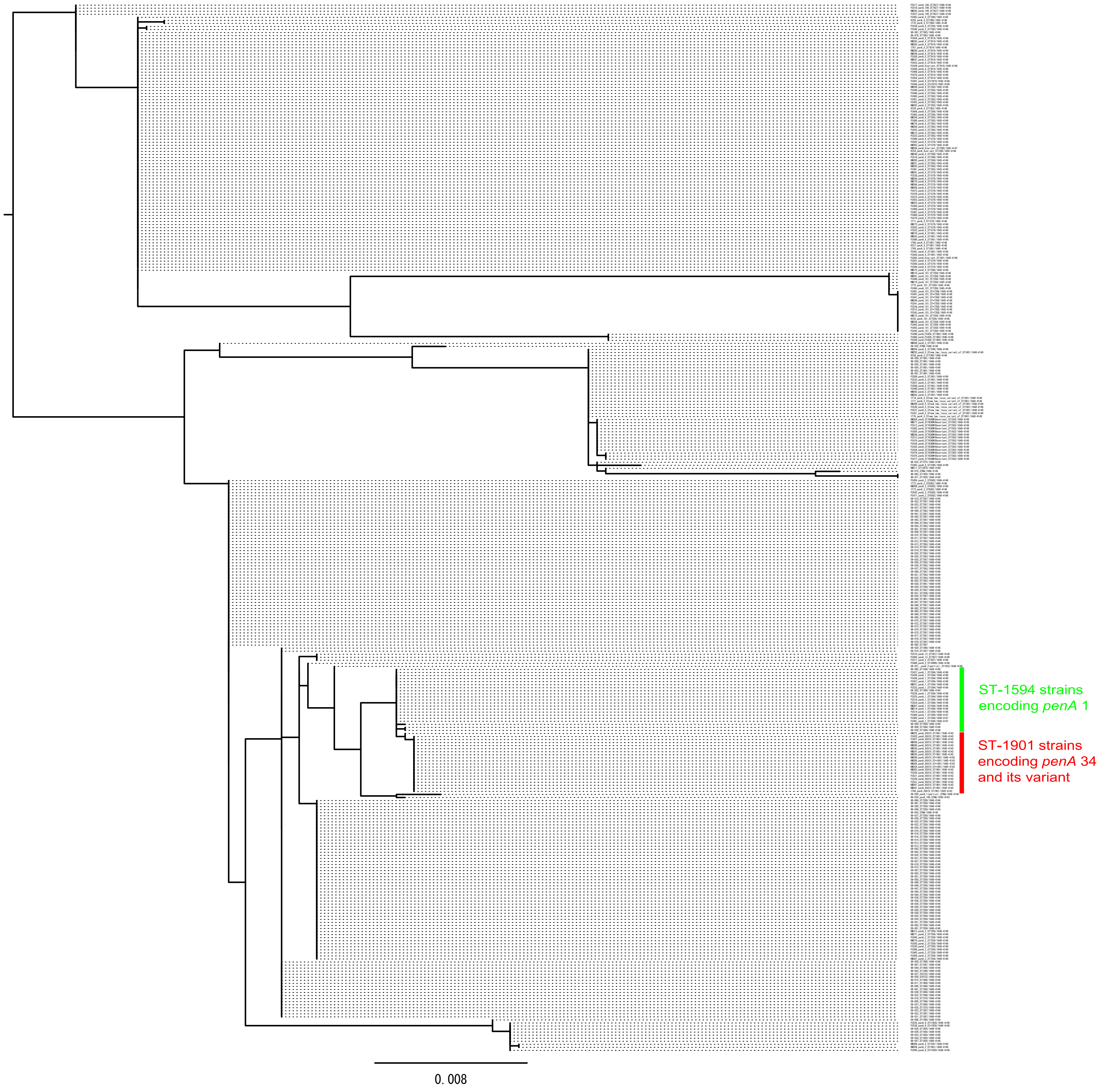

Fig S7. Maximum-likelihood tree of the recombined region (orange in Fig. 3). The tree includes 204 strains isolated through the genomic surveillance in 2015 in the Japanese prefectures of Kyoto and Osaka, and 140 strains isolated in 1996-1997 in the Japanese prefecture. Strain names with STs (and *penA* alleles for the 204 strains) are indicated at the right and searchable in this figure. If an asterisk is added to ST, it means a variant.
